# Mons pubis Sparganosis infection: A case report

**DOI:** 10.3892/mi.2025.287

**Published:** 2025-11-28

**Authors:** Jiang Zhu, Qiheng Li, Qingfeng Tang

**Affiliations:** 1 Department of Urology, People's Hospital of Xindu District, Chengdu, Sichuan 610599, P.R. China; 2 Department of Pathology, Chengdu Medical College Second Affiliated Hospital Nuclear Industry 416 Hospital, Chengdu, Sichuan 610066, P.R. China

**Keywords:** sparganosis, migration mode, infection, subcutaneous masses, pathology

## Abstract

Sparganosis is a key food-borne zoonosis and is typically endemic to the southeast coast of China. The present study reports a very rare case of sparganosis occurring in the mons pubis. The case described herein highlights the importance of considering subcutaneous sparganosis as a differential diagnosis in patients with subcutaneous masses. The disease manifests predominantly as subcutaneous nodules or intracranial mass lesions. Infection is primarily acquired by ingesting raw or undercooked amphibian or reptile flesh or by the consumption of water containing copepods. An accurate diagnosis and early treatment are crucial for the management of this infection.

## Introduction

Human sparganosis is a food-borne zoonosis caused by the plerocercoid larvae (sparganum) of tapeworms belonging to the genus *Spirometra*. Although human sparganosis is relatively infrequent globally, the majority of cases occur primarily in East and Southeast Asian countries due to dietary habits and traditional medical practices (1). The disease poses a critical public health concern due to its transmission through multiple pathways, including the ingestion of contaminated water, the consumption of raw or undercooked freshwater frogs or snakes, and the application of raw meat poultices to treat infections. Sparganosis is principally characterized by a slowly migrating subcutaneous nodule(s) and can invade the brain, eyes, breasts, spinal cord and subcutaneous tissues, often resulting in severe illness (2). In the first week following parasite infection, the host may develop an inflammatory cellular infiltrate. Tunnel-like structures appear 2 weeks thereafter, and fibroblast proliferation occurs 4 weeks following infection.

The present study reports an extremely rare case of sparganosis found in the mons pubis of a 26-year-old female patient who was immunocompetent. The purpose of the present case report was to enhance the understanding of sparganosis and emphasize that parasitic infection should be considered as a differential diagnosis when encountering a patient presenting with durable, wandering skin nodules.

## Case report

**Figure 1:**
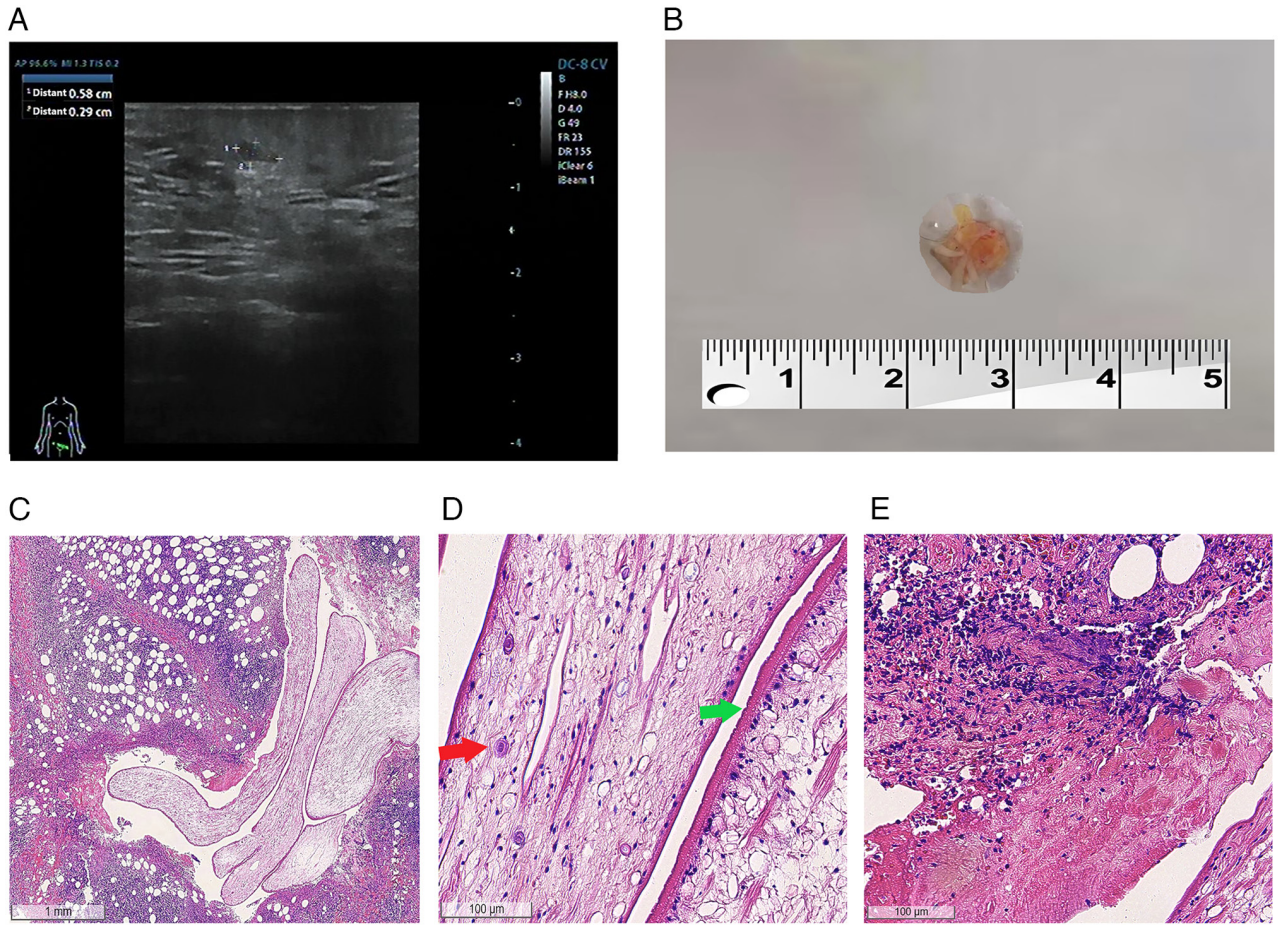
Detection of Spirometra mansoni sparganosis in the reported case. (A) Ultrasonography examination illustrating hypoechoic nodules 0.6x0.3 cm in size. (B) Surgical incision of the subcutaneous mass of the mons pubis, revealing suspected parasitic tissue. (C) Pathological examination of the subcutaneous mass showing larval tissue embedded in dense connective tissue (magnification, x40). (D) Higher magnification image illustrating the presence of typical histopathological features of cestodes, such as the highly folded eosinophilic noncellular tegument (green arrow) and the presence of calcareous corpuscles (red arrow) (magnification, x400). (E) Chronic granulomatous inflammation with foreign bodies associated with acute suppurative inflammation is observed in the tissues surrounding the parasite (magnification, x400). The sections depicted in panels C-E were stained with hematoxylin and eosin.

A 26-year-old Chinese female patient presented to People's Hospital of Xindu District, Chengdu, Sichuan, China, in November, 2023 with a subcutaneous nodule in the mons pubis. Of note, 2 months prior, the patient complained that subcutaneous nodules appeared on the abdomen, which were not treated due to her history of lipoma and normal skin temperature and color. These nodules disappeared spontaneously in ~10 days. However, a nodule was later found at the mons pubis that persisted for >1 month, prompting her to return to the hospital for treatment. During the course of the disease, there was no obvious pain in the nodule, and the patient did not suffer from headaches or nausea. The complete blood count from laboratory tests revealed an increased in peripheral eosinophilia. Routine blood tests were performed, including a complete blood count, hematocrit level and platelet count tests, liver function tests and renal function tests, all of which fell within the normal reference range; the results did not reveal anything uncommon. An ultrasonography revealed hypoechoic nodules detectable subcutaneously at the mons pubis mass, measuring ~0.6x0.3 cm in size, with clear borders and a regular morphology (Fig. 1A). No obvious blood flow signal was detected in the above lesions on color Doppler flow imaging (CDFI).

The nodule located on the mons pubis was completely surgically resected, and filamentous wormlike objects were found within the resected tissue. The precise length of the sample could not be determined due to fragmentation during extraction; however, it was >0.4 cm (Fig. 1B). A histological examination revealed parasites characterized by a deep-folded tegument and calcareous corpuscles, as well as peripheral inflammatory changes in the parasite body, accompanied by inflammatory cell infiltration. Hematoxylin and eosin (H&E) staining for all sections presented in Fig. 1C-E was independently performed by the Department of Pathology, People's Hospital of Xindu District, Chengdu, China. The detailed procedures were as follows: Tissues were fixed in formalin at room temperature for 24 h, followed by processing into paraffin blocks after sampling. Paraffin sections were cut at a thickness of 4 µm, which were then floated and flattened on warm water at 45˚C. The sections were retrieved onto glass slides and baked in an oven at 65˚C. For subsequent H&E staining, paraffin sections were first dewaxed and rehydrated. They were then stained with hematoxylin solution (MilliporeSigma) for 3-5 min, rinsed thoroughly with running water, and blued with warm water. Following this, the sections were stained with eosin solution (MilliporeSigma) for 2 min. Finally, the sections underwent dehydration, clearing, and mounting procedures before being observed under an optical microscope (LEICA DM3000; Leica Microsystems GmbH). In conjunction with the epidemiological history and the pathological examination, the patient was ultimately diagnosed with subcutaneous sparganosis (Fig. 1C). Subsequently, anti-sparganum IgG was detected in the serum of the patient using enzyme-linked immunosorbent assay (ELISA) (data not shown).

A pathological examination further revealed that the walls of these parasites were multilayered with a thick eosinophilic microvillous noncellular tegument, and numerous small clear vesicles were noted under the tegument. Moreover, numerous secretory canaliculi, subcutaneous cells and calcareous bodies were observed within the cytoplasm (Fig. 1D). Scolex, suckers, a digestive tract and reproductive organs were not present. A pathological examination of the largest lesion surrounding the worm revealed granulomatous inflammation and tunnel-like necrosis with eosinophilic, neutrophilic and lymphocytic infiltration (Fig. 1E). Sparganosis was confirmed based upon these results. The nodule was completely surgically resected, and wound healing proceeded without complications. No oral anthelminthic treatment was administered, and no subsequent lesions developed. Serological tests for anti-sparganum IgG were negative at 8 months following surgery, and no recurrence was observed.

## Discussion

Due to the constant occurrence of sparganosis in China, measures are required to prevent and control the disease. First, food safety and prevention measures for this disease should be emphasized and improved. The crucial point in preventing human sparganosis is to cease the consumption of raw or undercooked frog, snake and contaminated water, and the application of raw frog flesh or skin to open wounds.

Sparganosis is a zoonotic parasitic disease caused by the larvae (spargana) of the genus Spirometra, which is widely distributed globally and threatens human health (3,4). Humans serve as accidental secondary intermediate hosts, acquiring infection through the consumption of raw or undercooked meat from intermediate hosts, such as frogs or snakes, or by ingesting water contaminated with infected copepods. While cases have been sporadically documented across the globe, the disease is more prevalent in Asian regions, particularly in China, Korea, and Thailand (5,6). Sparganosis infections are primarily associated with the consumption of undercooked frogs or snakes, which serve as key sources of transmission. Following ingestion by humans, the larvae preferentially migrate into soft subcutaneous tissues; however, they may occasionally migrate into vital organs, such as the brain, spinal cord, or eyes, causing deleterious effects.

The clinical diagnosis of sparganosis involves a physical examination of cutaneous and other nodules, a computed tomography (CT) assessment of the location and dimension of the nodule, and magnetic resonance imaging (MRI) for the demonstration of a mass lesion or vasculopathy. Despite their obvious value, these procedures lack specificity and do not provide a definitive diagnosis (7). Laboratory methods for the diagnosis of sparganosis include the macro- and microscopic identification of spargana recovered through tissue biopsy, biochemical tests, serological assays and molecular techniques. In rare cases, next-generation sequencing has been used to identify some uncommon parasite species (8). The sparganum larvae are characterized as white, wrinkled and ribbon-shaped, varying from a few millimeters to 50 cm in length (4). Histopathological analysis reveals fibrous cystic tunnels containing live worms, eosinophilic granulomas, and intact worm structures exhibiting loose stroma, smooth muscle, secretory tegument and calcareous bodies. Biochemical tests may reveal pronounced peripheral eosinophilia (9). By contrast, the diagnosis of visceral or cerebral sparganosis poses significant challenges, and serological testing, particularly ELISA for anti-sparganum antibodies, serves as the primary diagnostic tool due to its high sensitivity and specificity.

In the case in the present study, the involvement of the mons pubis was extremely rare. Precisely due to its unique location, the initial diagnosis invariably leaned toward gynecological conditions. Relevant differential diagnoses were excluded through examinations, including HPV-DNA testing, ELISA and tissue biopsy. The presence of subcutaneous mobile masses in the medical history suggested that parasitic infection should be highly considered.

Treatment is primarily based on the surgical removal of the larva. No medical treatment standard has been defined for patients with masses not suitable for surgical resection, and high-dose praziquantel (repeated cycles of 50-75 mg/kg body weight/day for 10 days) has been used to treat infection (10,11). To the best of our knowledge, mons pubis sparganosis has rarely been reported. In the case described herein, the patient underwent successful surgical removal. Following treatment, the patient exhibited a reduction in eosinophil levels, and serological testing for anti-sparganum IgG yielded negative results, indicating the efficacy of surgical intervention for mons pubis sparganosis. The patient recovered well following the procedure.

In conclusion, the present case report, the young female patient had a subcutaneous nodule of the mons pubis. Sparganosis was diagnosed by ELISA and tissue biopsy. This rare case of sparganum infection is a reminder that when patients presenting with persistent and migratory skin nodules are encountered, regardless of the location, parasitic infection should be considered as a differential diagnosis. Furthermore, it is considered that health education on avoiding the consumption of undercooked frogs and unboiled water is of utmost importance in preventing parasitic infections.

## Data Availability

The data generated in the present study may be requested from the corresponding author.
